# Non-invasive brain stimulation in neurorehabilitation: local and distant effects for motor recovery

**DOI:** 10.3389/fnhum.2014.00378

**Published:** 2014-06-27

**Authors:** Sook-Lei Liew, Emilliano Santarnecchi, Ethan R. Buch, Leonardo G. Cohen

**Affiliations:** ^1^Human Cortical Physiology and Neurorehabilitation Section, National Institute of Neurological Disorders and Stroke, NIHBethesda, MD, USA; ^2^Department of Medicine, Surgery and Neuroscience, University of SienaSiena, Italy; ^3^Center for Neuroscience and Regenerative Medicine, Uniformed Services University of Health SciencesBethesda, MD, USA

**Keywords:** non-invasive brain stimulation, transcranial direct current stimulation (tDCS), transcranial magnetic stimulation, neurorehabilitation, stroke

## Abstract

Non-invasive brain stimulation (NIBS) may enhance motor recovery after neurological injury through the causal induction of plasticity processes. Neurological injury, such as stroke, often results in serious long-term physical disabilities, and despite intensive therapy, a large majority of brain injury survivors fail to regain full motor function. Emerging research suggests that NIBS techniques, such as transcranial magnetic (TMS) and direct current (tDCS) stimulation, in association with customarily used neurorehabilitative treatments, may enhance motor recovery. This paper provides a general review on TMS and tDCS paradigms, the mechanisms by which they operate and the stimulation techniques used in neurorehabilitation, specifically stroke. TMS and tDCS influence regional neural activity underlying the stimulation location and also distant interconnected network activity throughout the brain. We discuss recent studies that document NIBS effects on global brain activity measured with various neuroimaging techniques, which help to characterize better strategies for more accurate NIBS stimulation. These rapidly growing areas of inquiry may hold potential for improving the effectiveness of NIBS-based interventions for clinical rehabilitation.

## Introduction

Stroke is a leading cause of serious long-term adult disability around the world. Recovery of motor function remains highly variable despite standardized rehabilitation programs (Kwakkel et al., 2003; Go et al., 2013). The study of the mechanisms underlying recovery of motor function after stroke has been difficult due to the heterogeneity among individual lesion profiles, the severity of motor impairment and the differences in plasticity processes depending on the time passed since the ictal event.

Non-invasive brain stimulation (NIBS) has been explored as a possible technical adjuvant of customarily used neurorehabilitative treatments. NIBS, which employs electrical or magnetically-induced currents to stimulate the brain through the scalp, can temporarily excite or inhibit activity in target brain regions. In this review, we first introduce the use of NIBS in basic science and clinical neuroscience, focusing on the two most commonly used NIBS techniques (transcranial magnetic stimulation, *TMS*, and transcranial direct current stimulation, *tDCS*). We then delve into recent work exploring the effects of local application of NIBS on activity under the stimulating site and in distant brain regions. We discuss the evidence for the application of NIBS techniques in motor rehabilitation and provide a map of possible future research directions, including the combined use of NIBS with neuroimaging techniques, and the use of transcranial random noise stimulation and transcranial alternating current stimulation, among others.

## Background

**Figure 1:**
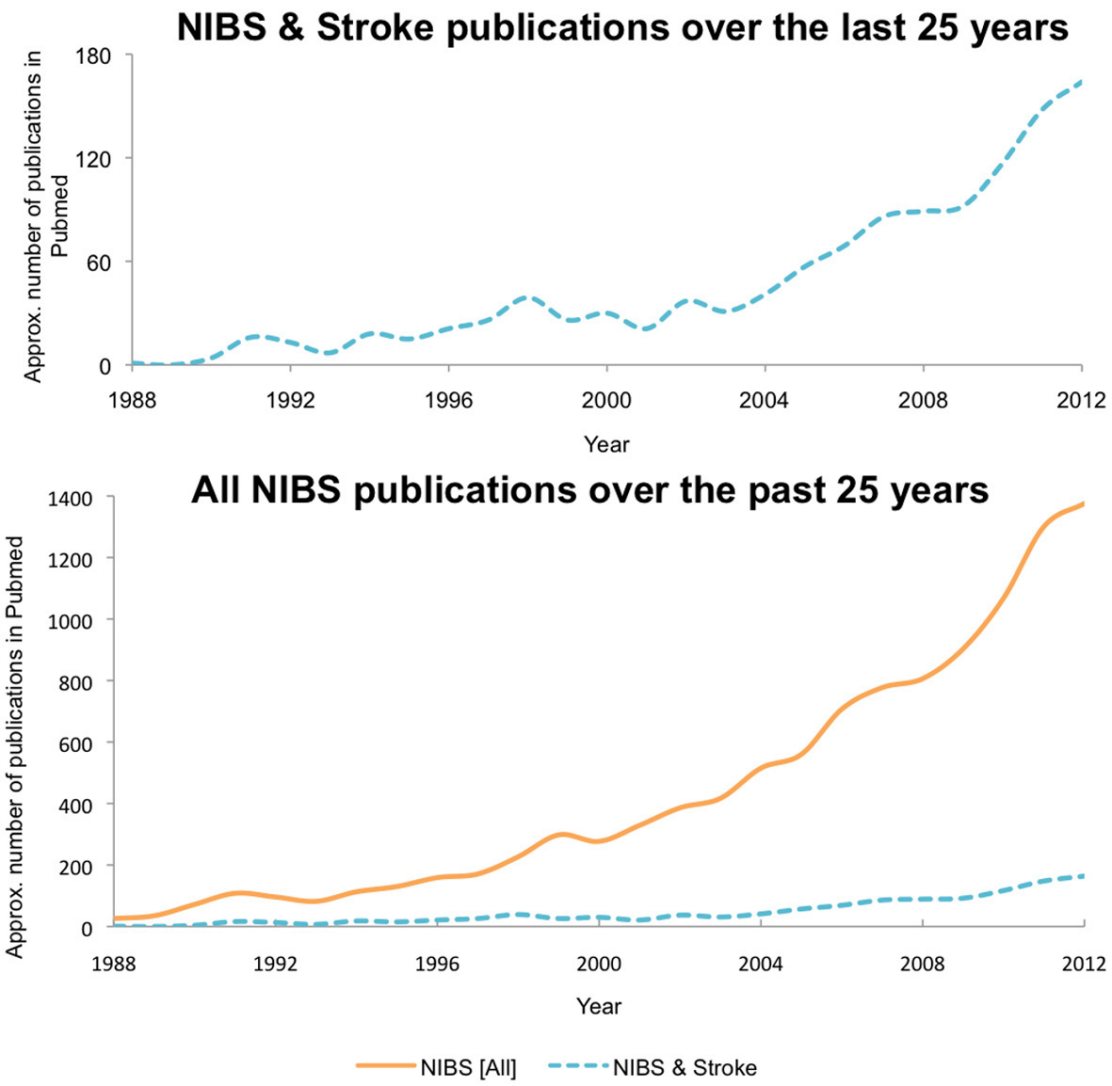
**NIBS publications**. Graph depicting exponential growth in the number of publications on NIBS from 1988 to 2012, with NIBS publications specific to stroke depicted at the top, and NIBS publications specific to stroke shown in the context of the general NIBS field at bottom.

Early studies of “therapeutic electricity” can be traced back to the late 1800s. Since then, NIBS applications have been used in a variety of settings (for reviews, see Priori, 2003; Wagner et al., 2007a,b; Schlaug and Renga, 2008). Scientific research and public awareness of these techniques has increased greatly over the last few decades. While only a handful of papers were published on the topic in 1988, almost 1400 papers were published in 2012 alone (see Figure 1).

In contrast, the use of NIBS in neurorehabilitative settings has more recently taken off, starting in the mid-2000s (Elbert et al., 1981; Ward and Cohen, 2004; Hummel et al., 2005; see Figure 1). Currently, the most common NIBS techniques are TMS and transcranial electric stimulation (tES; for a recent review, see Dayan et al., 2013). NIBS is thought to modulate neural activity via differing mechanisms, including the induction of LTP-like protocols (Ziemann and Siebner, 2008; Fritsch et al., 2010; Muller-Dahlhaus et al., 2010; Ziemann, 2011). It has been proposed that modulation of these mechanisms induce motor plasticity, contributing to motor learning (Reis et al., 2009; Censor et al., 2010; Fritsch et al., 2010; Buch et al., 2011; Dayan and Cohen, 2011; Schambra et al., 2011; Conde et al., 2013) and secondarily impacting neurorehabilitative processes (Dimyan and Cohen, 2010, 2011).

## Types of NIBS

**Figure 2:**
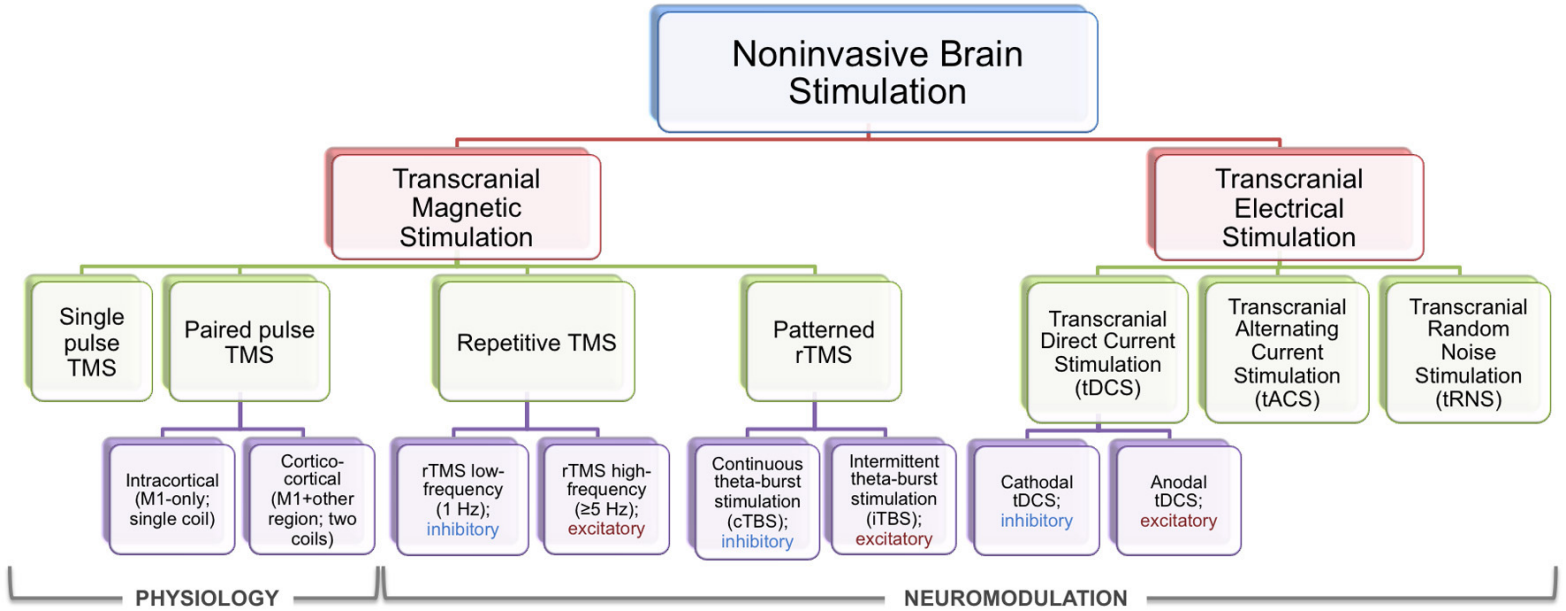
**NIBS schematic**. Chart depicting the general breakdown of NIBS techniques, focusing on TMS and tES. Types of TMS and tES paradigms are describe, and the divide between physiology and neuromodulatory functions is depicted. Inhibitory and excitatory neuromodulatory techniques are also labeled.

NIBS techniques have been tested in a wide array of research and clinical settings (Dayan and Cohen, 2011; Song et al., 2011; Ziemann, 2011; Censor et al., 2013; Sandrini and Cohen, 2013; Vidal-Dourado et al., 2014), and the testing of NIBS to modulate learning and memory processes has attracted particular attention in the last few years (for reviews, see Tanaka et al., 2011; Kandel et al., 2012; Sandrini and Cohen, 2013). While there is wide variation in stimulation protocols, traditional TMS and tDCS mechanisms and protocols are discussed briefly here (see Figure 2 for a summary diagram; see Box 1 for safety considerations).

Box 1 **Safety Considerations for TMS and tDCS**

***Safety considerations for TMS***

Apart from general safety considerations regarding tissue heating, magnetization of ferromagnetic objects, and magnetic field exposure for both subjects and operators, consideration must be given to potential side effects of TMS, which consist primarily of the rare induction of seizures, as well more common effects like local transient pain, headaches, and discomfort (Rossi et al., 2009). Consequently, while there are no specific concerns about single and paired pulse TMS applications, rTMS and patterned rTMS deserve specific attention in terms of the number of stimuli delivered per unit time. Generally speaking, the safety of high-frequency rTMS protocols is usually assured by including periods of no stimulation between shorter periods of rTMS. TBS protocols are usually applied by replicating the original protocol published by Huang et al. (2005), consisting of 3 pulses at 50 Hz applied at 5 Hz for 20 or 40 s, in the case of cTBS. In contrast, iTBS is obtained by conducting 2-s periods of cTBS, each separated from one another by 8 s. It must be noticed that there are almost an infinite number of combinations for such protocols, with even small changes possibly having strong impacts on both the effects and safety of such protocols. Thus, general guidelines for rTMS delivery should be always checked, particularly when applied in clinical settings. Additionally, it should be considered that the effects of these techniques present interindividual differences.

***Safety considerations for tDCS***

Compared to TMS, tDCS is relatively safer and easier to use. A vast literature supports the use of low-intensity transcranial stimulation as safe for use in humans, with only rare and relatively minor adverse effects, such as mild tingling of the scalp, minor fatigue, or itching of the scalp (Poreisz et al., 2007) and no effects over serum levels of molecular markers of neuronal injury such as neuron-specific enolase (Nitsche et al., 2003b) or N-acetyl-aspartate (Rango et al., 2008). It must be noticed that all the aforementioned effects of tDCS are strongly dependent on current density, electrode positioning, and stimulation duration. While differences in such parameters may be of interest for their consequences over observed behavioral responses, they must also be taken into account for safety purposes. For instance, caution should be used during monopolar stimulation with extracephalic references due to the hypothetical stimulation of brainstem regions, thus possibly modulating sympathetic outflow (Cogiamanian et al., 2010). However, such findings are still a matter of debate. Most importantly, anatomical changes due to central nervous system pathology can significantly modify the current distribution induced by tDCS. For instance, in subjects with stroke, the affected cortical area is usually replaced by cerebrospinal fluid, which has a high conductance, and current can accumulate on the edges of cortical stroke lesions (Wagner et al., 2007a,b).

### TMS

First introduced by Barker et al. (1985), TMS used within international safety guidelines is safe and non-invasive (Kobayashi et al., 2003; Rossini and Rossi, 2007). TMS produces a time-varying magnetic field at that flows perpendicular to the stimulating coil, which then induces electric currents that are generally parallel to the coil in the underlying cortical tissue. The specific protocol and magnetic coil design allows TMS to stimulate cortical tissues at variable depths beneath the scalp (Cohen et al., 1998).

TMS can be used to assess neurophysiological processes and influence brain function via application of single, paired, or repetitive stimulation. In single-pulse TMS (*spTMS*), one single stimulus is applied, for example, over the primary motor cortex (M1; Reis et al., 2008). When the intensity of the stimulus is strong enough (suprathreshold), it will induce a measurable electromyographic (EMG) response in target hand muscles contralateral to the stimulated M1, known as a motor-evoked potential (MEP). spTMS may be used to map M1 corticospinal outputs, study central motor conduction time, and investigate causal chronometry in brain-behavior relations (for a review, see Dayan et al., 2013). Due to the relative simplicity of recording with surface EMG electrodes, spTMS-induced MEPs have become a routine procedure in clinical neurophysiology for assessing the functional integrity of corticospinal and corticobulbar motor pathways in a wide range of neurological disorders (Rossini and Rossi, 2007). Paired (ppTMS) or triple-pulse TMS (tpTMS) utilize one or more conditioning stimuli applied prior to a suprathreshold M1 (test) stimulus that induces a measurable MEP (Groppa et al., 2012). This technique can be used to investigate intra- or cortico-cortical neuronal interactions depending on the precise latency and intensity (sub- or supratheshold) of the conditioning pulses, and depending whether they are applied to the target region or to an interconnected brain region. For example, ppTMS applied to M1 has been used to investigate different aspects of local interneuron dynamics with the resulting effect of the conditioning pulse on the output MEP demonstrating intracortical facilitation (ICF) or inhibition (ICI), depending on the latency of stimulation (Chen et al., 1998; Cohen et al., 1998; Boroojerdi et al., 2000). ppTMS can be applied to different sites to evaluate the effects of a stimulus on one region over the excitability of a different brain region. In this form, ppTMS can test cortico-cortical connectivity between two different regions. For example, connectivity can be assessed between homologous regions of both M1s (with this effect referred to as interhemispheric inhibition; Di Lazzaro et al., 1999; Murase et al., 2004; Duque et al., 2005), between premotor cortex and M1, between dorsolateral prefrontal cortex (DLPFC) and M1, between the posterior parietal cortex and M1, and between the cerebellum and M1 (Oliveri et al., 2005; Koch et al., 2007; Daskalakis et al., 2008; Buch et al., 2010). This work provides insight into the causal relationship of prefrontal, frontal, and parietal inputs on M1 corticospinal output within motor behavioral contexts such as prehension, action selection, and action reprogramming. Investigations of these dynamics in patient groups, such as chronic stroke, have revealed relationships between altered cortico-cortical interactions and behavioral deficits (for example, see Murase et al., 2004; Nowak et al., 2009).

Repetitive TMS (*rTMS*) can also be used as a neuromodulatory tool. Low-frequency rTMS (≤1 Hz) can be used to transiently perturb the stimulated brain region inducing a so-called “virtual lesion” (Pascual-Leone et al., 1999). This form of inhibitory rTMS represents an *in-vivo* non-invasive method available for demonstrating the causal influence of a given cortical region or its interconnected network on specific behaviors (Chen et al., 1997; Cohen et al., 1997; Walsh and Cowey, 2000). rTMS induces frequency- and intensity-specific after-effects, with low-frequency stimulation (≤1 Hz; Chen et al., 1997) inducing a decrease in cortical excitability as described previously, while high-frequency stimulation (≥5 Hz) results in an increase in MEP amplitude, increasing activation within the region for at least 30 min (Rossi et al., 2009). Depending on the specific stimulation protocol used, the neuromodulatory effects of rTMS can outlast the stimulation period by several minutes to hours. Paired associative stimulation (PAS) is a related technique that involves application of a peripheral nerve stimulus followed by a TMS pulse at varying interstimulus intervals. Pairs are applied at very low-frequency (0.1 Hz) to M1 and to a peripheral nerve (Stefan et al., 2000, 2002; Wagner et al., 2007a,b). By varying the inter-stimulus intervals, PAS can induce potentiation or inhibition of M1 corticospinal output lasting for up to 90 min. A modified version of this protocol has been developed to investigate the induction of associative plasticity within cortico-cortical pathways (Rizzo et al., 2009; Buch et al., 2011).

Another form of rTMS is patterned rTMS. It consists of the repetitive application of short rTMS bursts at a high stimulation frequency. The most common paradigm is theta burst stimulation (TBS, continuous cTBS or intermittent iTBS), in which short bursts of 50 Hz rTMS are applied at a rate in the theta range (5 Hz) (Huang et al., 2005). As with low and high-frequency rTMS, cTBS, and iTBS induce cortical depression and facilitate corticospinal excitability, respectively, in healthy subjects for up to 70 min. When applied to prefrontal areas, it may influence memory processes like reconsolidation of episodic memories (Sandrini et al., 2013). Of note, the effects of these different techniques on motor cortical excitability present substantial interindividual differences, the origin of which are under investigation. The use of this technique in clinical populations thus requires further work and a careful approach (Ridding and Rothwell, 2007).

### tDCS

tDCS is applied using a battery-powered direct current (DC) generator connected to two relatively large anodal and cathodal sponge-enclosed rubber electrodes (20–35 cm^2^ in area) positioned over the scalp. It is thought that low amplitude currents (ranging from 0.5 to 2.0 mA) applied at the scalp can partially penetrate and reach cortical tissues (Datta et al., 2009). In contrast to TMS, tDCS does not result in the induction of action potentials. tDCS seem to modify the threshold for discharge of cortical neurons (Nitsche and Paulus, 2001; Priori, 2003). As a reference point, the magnitude of tDCS stimulation (0.079–0.20 A/m2) is far below the range of action potential thresholds (22–275 A/m2).

tDCS can modulate cortical excitability in a polarity-dependent fashion. While anodal stimulation increases cortical excitability, cathodal stimulation is thought to decrease it. It should be noted though that these effects, as those of facilitatory and inhibitory TMS, exhibit high interindividual variability (Ridding and Rothwell, 2007) and depend on the activity levels of the stimulated tissues (Silvanto et al., 2008). Both produce after-effects lasting 30–40 min, following 15–30 min of stimulation, with the after-effects strongly dependent on the duration and intensity of the stimulation (see Nitsche and Paulus, 2001). In addition, the direction of such polarization strictly depends on the orientation of axons and dendrites in the induced electric field. While tDCS has been initially shown to modulate activity in both the motor and visual cortices (Nitsche and Paulus, 2011), recent evidence has suggested that it is also efficacious in modulating higher-order cognitive processes through its applications over prefrontal and parietal regions (Nitsche et al., 2012; Monti et al., 2013; Santarnecchi et al., 2013).

Special consideration should be given to the placement of the electrodes and the focality of tDCS interventions. Newer tDCS montages include bipolar and monopolar scalp stimulation, with the former consisting of both cathode and anode placed on the scalp surface, while the latter positions the “active” electrode on the scalp, with the “reference” placed on an extracephalic target (shoulder, leg, arm, etc.; Schambra et al., 2011). Different electrode configurations may result in different patterns of current spreading over the scalp and consequently on the cortex; it is feasible that the typical “reference” position over the supraorbital region may produce undesired stimulation in non-target regions, thus newer monopolar stimulation montages attempt to avoid this problem (DaSilva et al., 2011). In addition, it has been proposed that high-resolution tDCS may improve this form of stimulation's focality (high-definition tDCS, or HD-tDCS; Datta et al., 2009). From an instrumental point of view, HD-tDCS uses multiple sites of anodal and cathodal stimulation to target a specific region. While substantial work is under way to model the fields induced by these different montages, clear behavioral or physiological data is lacking on the differences between these approaches.

While tDCS-induced changes in cortical excitability have been related to changes in the underlying cortical neuronal activity, less is known about the specific mechanisms mediating these effects. It has been reported that carbamazepine, dextromethorphan, and the calcium channel blocker flunarizine diminish the effects of anodal tDCS on motor cortical excitability (Nitsche et al., 2003a). On the contrary, the partial NMDA agonist D-cylcoserine prolongs the effects of anodal tDCS on cortical excitability (Nitsche et al., 2004). Anodal tDCS applied to a slice preparation of rodent M1 induced LTP-like effects. This effect was NMDA-receptor dependent and mediated by secretion of brain-derived neurotrophic factor (BDNF; Fritsch et al., 2010). Overall, these findings suggest that the magnitude of membrane polarization, the conductance of sodium and calcium channels, the magnitude of NMDA receptor activity as well as BDNF secretion contribute to different extents to the tDCS after-effects. These findings open the possibility of pharmacologically modulating tDCS effects.

tDCS has also been tested in small clinical trials evaluating corticospinal excitability, neurophysiological changes, and the modulation of behavioral variables in neurological and psychiatric diseases such as depression, chronic pain, epilepsy, neuropsychiatric disorders, and stroke, with mixed results (for reviews, see Nitsche and Paulus, 2011; Rothwell, 2012).

## Network effects of NIBS

Recently, a wealth of studies have begun to demonstrate that brain stimulation leads not only to local changes in activity under the stimulated coil or electrodes, but also to distant changes in interconnected brain regions throughout the brain (for reviews, see Siebner et al., 2009; Siebner and Ziemann, 2010).

Successful behavior requires the concerted action of multiple brain regions. Neuroimaging studies started to provide important information on the activity of these different networks. In this setting, regions in communication with one another are thought to be highly synchronized (Biswal et al., 1995; Fries, 2005). Interregional connectivity can be analyzed as simple correlations between regions' activations and phase-locked coherence in neural oscillations, or can be modeled with more complex approaches that include *a priori* hypotheses (e.g., using dynamic causal modeling, *DCM*). It is now known that patterns of functional connectivity are predictive of successful motor behaviors and motor recovery in healthy individuals and in patients with stroke (for reviews, see Grefkes and Fink, 2011, 2012). Thus, while individual regions perform specific functions, the sharing of this information amongst a wide array of interconnected regions is critical for successful behavior. Given this information, the ability of NIBS to modulate activity locally and in interconnected networks seems valuable. There is substantial research activity in this area.

### TMS and connectivity

Early studies demonstrated it is possible to evaluate changes in brain activity after TMS using single-photon emission computerized tomography (SPECT) (Shafran et al., 1989; Dressler et al., 1990). Several groups performed similar evaluations using positron emission tomography (PET) while participants underwent TMS stimulation (Fox et al., 1997; Paus et al., 1997; Paus, 1999). Other studies evaluated neurophysiological rather than blood flow changes induced by TMS using electroencephalogram (EEG) (Amassian et al., 1992). In the late 1990s, Bohning et al. (1997, 1998) demonstrated the feasibility of recording blood-oxygen-level dependent (BOLD) signal activity changes using fMRI in close temporal proximity to TMS. This early work documented local and distant changes in regional cerebral blood flow and in physiological activity associated with focal TMS stimulation. In the two decades since this pioneering work, researchers have developed new paradigms of combined brain imaging and brain stimulation to explore the effects of focal stimulation on global brain activity (see Table 1).

**Table 1:** **Studies showing the effects of TMS on neural connectivity**.

**Stimulation site**	**Stimulation type**	**Neuroimaging technique**	**Population**	**Connectivity increases**	**Connectivity decreases**	**Behavioral result**	**References**
Left M1	High-frequency rTMS (3.125 Hz), suprathreshold	fMRI	Healthy volunteers	M1/S1, SMA, dorsal premotor cortex, cingulate motor area, putament, thalamus			Bestmann et al., 2004
Left M1	High-frequency rTMS (3.125 Hz), subthreshold	fMRI	Healthy volunteers	SMA, dorsal premotor cortex, cingulate motor area, putamen, thalamus (but at a lower intensity)			Bestmann et al., 2004
Left M1	High-frequency rTMS (4 Hz), subthreshold	fMRI	Healthy volunteers	SMA, bilateral premotor cortex	Right M1/S1		Bestmann et al., 2003
Left M1	High-frequency rTMS (4 Hz), suprathreshold	fMRI	Healthy volunteers	Left M1/S1, SMA	Right M1/S1		Bestmann et al., 2003
Right M1	Low-frequency rTMS (1 Hz)	fMRI	Healthy volunteers		Decreased connectivity between right M1 and SMA, bilateral anterior cerebellum, right dorsal striatum, and left M1	Decreased SMA activity corresponded with decreased motor memory modificiation	Censor et al., 2013
Left dorsal premotor cortex (PMd)	High-frequency rTMS (3 Hz), suprathreshold	fMRI	Healthy volunteers	Left PMd, left premotor ventral (PMv), right PMd, bilateral PMv, SMA, somatosensory cortex, cingulate motor area, left posterior temporal lobe, cerebellum, caudate nucleus			Bestmann et al., 2005
Left dorsal premotor cortex (PMd)	High-frequency rTMS (3 Hz), subthreshold	fMRI	Healthy volunteers	Bilateral PMv, SMA, bilateral auditory cortex, bilateral thalamus, bilateral cingulate gyrus			Bestmann et al., 2005
Contralesional PMd	High-frequency rTMS (11 Hz), suprathreshold	fMRI	Chronic stroke patients	Increased activity in ipsielsional sensorimotor cortex		Greater ipsilesional sensorimotor cortex activity after rTMS to contralesional PMd correlated with greater motor impairment	Bestmann et al., 2010
Ipsilesional M1	High-frequency rTMS (10 Hz), subthreshold	PET	Chronic stroke patients	Altered effective connectivity between ipsilesional M1, basal ganglia, thalamus; altered interhemispheric connectivity		Ipsilesional TMS response covaries with improvement after movement therapy	Chouinard et al., 2006
Contralesional M1	Low-frequency rTMS (1 Hz)	fMRI	Subacute stroke patient	Increased coupling between ipsilesional SMA and M1		Inhibitory contralesional TMS improved motor performance of paretic hand; decreased influences of contralesional M1 after rTMS correlated with motor improvement	Grefkes et al., 2010

In healthy volunteers, Bestmann and colleagues demonstrated that suprathreshold high-frequency rTMS stimulation over M1 induces BOLD signal changes in distant cortical and subcortical regions, including the primary sensorimotor, supplementary and premotor cortices, as well as in the putamen and thalamus (Bestmann et al., 2004). Consistently, high-frequency suprathreshold rTMS over M1 enhanced connectivity with the supplementary motor area (SMA) (Bestmann et al., 2003). More recently, it was shown that low-frequency inhibitory rTMS over M1 also modified connectivity between M1, SMA, and the anterior cerebellum, and more importantly, showed that modulation of such connectivity correlated with the ability of healthy humans to modify a previously consolidated motor memory (Censor et al., 2013).

Application of rTMS over regions other than M1 also modulates functional activity. Suprathreshold rTMS over the left dorsal premotor cortex (PMd) for example increases BOLD signal locally, under the stimulating coil, and in distant regions like the right PMd, bilateral ventral premotor cortex, SMA (Bestmann et al., 2005).

In patients with chronic stroke, subthreshold rTMS over the ipsilesional M1 modulates interhemispheric and effective connectivity between this region, the basal ganglia and the thalamus (Chouinard et al., 2006). Inhibitory rTMS over the contralesional M1 resulted in increased connectivity between the ipsilesional M1 and SMA (Grefkes et al., 2010). These results suggest that reducing excitability and connectivity of the contralesional M1 may result in increased connectivity of the ipsilesional M1. The finding that modulation of ipsilesional and contralesional M1 effective connectivity correlated with motor function in these patients (Grefkes et al., 2010), in concordance with Chouinard et al. (2006) work, is suggestive of a causal link between changes in connectivity and behavior.

Stimulation of the contralesional PMd in chronic stroke patients induced stronger connectivity between this region and the ipsilesional primary sensorimotor cortex in individuals with greater motor impairments (Bestmann et al., 2010), suggesting that contralesional influences from regions other than M1 are also relevant to behavior, particularly for patients with greater motor impairment. Future work is needed to examine these effects in greater detail.

Altogether, these studies suggest that facilitatory stimulation of ipsilesional M1 increases M1-SMA functional connectivity while inhibitory stimulation of contralesional M1 decreases contralesional but strengthens ipsilesional connectivity—a pattern that is associated with improved motor performance (Ward et al., 2003; Rehme et al., 2011). Additionally, stimulation of regions other than M1 also induces substantial connectivity changes in interconnected brain regions. See Bestmann et al. (2008), Ruff et al. (2009), Ferreri and Rossini (2013) for additional information on this issue.

### tDCS and connectivity

**Figure 3:**
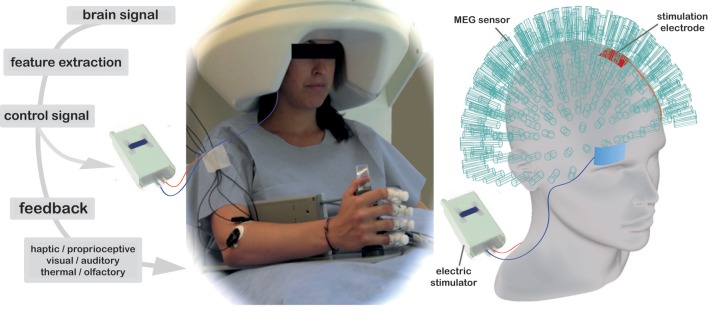
**Example of combined tDCS, MEG, and BCI experimental setup**. This design uses a 275-sensor whole-head MEG to record neuromagnetic brain activity during tDCS stimulation, with electrodes placed in the classic unilateral M1 montage (anode placed above the area of the right M1 and reference electrode above the left supraorbital area). This set-up is used in conjunction with BCI visual feedback in the form of a computer game and sensorimotor feedback via a robotic hand orthosis that opened as target oscillations increased. Image courtesy of S. Soekadar (Soekadar et al., under review).

tDCS also induces changes in connectivity between different brain regions, both at rest and during task performance. Initial evaluations of the influence of tDCS on cortical connectivity have primarily focused on the primary motor cortex (M1) and the DLPFC in healthy individuals (see Table 2). Functional connectivity before, during, and after tDCS application has been studied with EEG (for a review, see Miniussi et al., 2012), fMRI, arterial spin labeling (ASL) and, most recently, magnetoencephalography (MEG) (see Figure 3, Soekadar et al., 2013a,b).

**Table 2:** **Studies showing the effects of tDCS on neural connectivity**.

**Anodal stimulation site**	**Cathodal stimulation site**	**Neuroimaging technique**	**Task**	**Population**	**Connectivity increases**	**Connectivity decreases**	**Behavioral result**	**References**
Left M1	Right frontopolar cortex	EEG	Voluntary hand movements	Healthy volunteers	Increased intrahemispheric connectivity; increased connectivity patterns in left premotor, motor, sensorimotor regions in high- gamma 60–90 Hz range; increased synchrony in frontal and parieto-occipital regions in low-frequency (alpha and below) bands	Decreased interhemispheric connectivity		Polania et al., 2011a
Left M1	Right frontopolar cortex	EEG	Resting	Healthy volunteers	Increased synchronization within frontal electrodes in theta, alpha, and beta bands			Polania et al., 2011a
Left M1	Right frontopolar cortex	fMRI	Resting	Healthy volunteers	Increased coupling between left thalamus and Ml; increased connectivity between left caudate nucleus and parietal cortex			Polania et al., 2012b
Right frontopolar cortex	Left M1	fMRI	Resting	Healthy volunteers		Decreased coupling between left M1 and right putamen		Polania et al., 2012b
Left M1	Right frontopolar cortex	fMRI	Resting	Healthy volunteers	Increased nodal minimum path length in left sensorimotor cortex (less distant functional connectivity); increased coupling between left sensorimotor cortex and premotor and superior parietal areas			Polania et al., 2011b
Left M1	Right frontopolar cortex	fMRI	Voluntary hand movements	Healthy volunteers		Decreased activity in SMA during finger tapping with anodal tDCS compared to no stimulation		Antal et al., 2011
Left M1	Right frontopolar cortex	fMRI	Resting	Healthy volunteers	No significant effects	No significant effects		Antal et al., 2011
Left M1	Right frontopolar cortex	EEG	Resting	Healthy volunteers	Increase in power density of low frequency oscillations (theta, alpha)		Increased corticospinal excitability as indexed by MEP amplitude, and increased cortical reactivity	Pellicciari et al., 2013
Right frontopolar cortex	Left M1	EEG	Resting	Healthy volunteers	Increase in power density of low frequency oscillations (theta, alpha)		Decreased corticospinal excitability as indexed by MEP amplitude, and decreased cortical reactivity	Pellicciari et al., 2013
Right M1	Left M1	fMRI	Resting	Healthy volunteers	Increased connectivity between right Ml, PMd, bilateral SMA, and prefronal cortex			Sehm et al., 2012
Right M1	Right frontopolar cortex	fMRI	Resting	Healthy volunteers	Increased connectivity in left frontotemporal, bilateral pareital, and right cerebellar regions			Sehm et al., 2012
Right M1	Left M1	fMRI	Resting	Healthy volunteers	Increased intracortical connectivity	Decreased interhemispheric connectivity		Sehm et al., 2013
Left dlPFC	Right frontopolar cortex	fMRI	Resting	Healthy volunteers	Increased connectivity in the frontal component of the default mode network and bilateral frontoparietal networks			Keeser et al., 2011
Left dlPFC	Right frontopolar cortex	fMRI	Resting	Healthy volunteers	Increased functional connectivity between prefrontal and parietal regions	Decreased spatial robustness of default mode network		Pena-Gomez et al., 2012

Polania and colleagues demonstrated that tDCS applied over M1 influences cortical connectivity measured with EEG, with effects more evident when studying connectivity during voluntary hand movements than during rest (Polania et al., 2011a,b, 2012b). Anodal tDCS over left M1, with the cathode positioned over the contralateral supraorbital area increased synchronization in alpha and lower frequency bands in frontal and parieto-occipital regions, and in the high gamma frequency (60–90 Hz) band in motor-related regions (Polania et al., 2011a) during voluntary hand movements, with fewer changes during rest (Polania et al., 2011a). The same group studied the influence of tDCS on activity measured with fMRI, which consistently was more evident during hand movements than at rest (Antal et al., 2011). Another EEG study showed that anodal tDCS over left M1 during rest in healthy volunteers only increased the power density of low frequency oscillations (theta, alpha; Pellicciari et al., 2013). These results suggest that substantial changes in brain activity associated with tDCS are augmented by its combination with performance of an active behavioral task, as predicted from basic science studies (Fritsch et al., 2010).

The effects of tDCS on fMRI connectivity have also been studied using a graph theoretical approach. This analytical tool showed that anodal tDCS over M1 reduced the functional connectivity between the stimulated M1 and more distant regions but increased connectivity between the stimulated M1 and premotor and superior parietal regions (Polania et al., 2011b). In a different study, these authors demonstrated that anodal tDCS over M1 also increases connectivity between the stimulated region and subcortical structures on the same hemisphere, including the ipsilateral thalamus (Polania et al., 2012a,b). These findings are supported by Stagg et al. (2013), who demonstrated increases in perfusion MRI during anodal tDCS in regions anatomically-interconnected to the stimulated site. Thus, tDCS likely increases blood perfusion in the target site as well as in anatomically interconnected networks. While still speculative, these studies suggest that increasing M1 excitability through anodal tDCS exerts its greatest effects in high frequency bands during active task performance, and reduces distant connectivity, increasing local, intrahemispheric connectivity (both cortical and subcortical). Stimulation during rest appears to primarily influence low frequency bands, such as theta and alpha bands, while stimulation during active movement may additionally influence high gamma bands (Polania et al., 2011a, 2012b; Pellicciari et al., 2013).

Studies of effects of tDCS on cortical connectivity also examined the use of different stimulating montages. A direct comparison of the effects of bilateral (with the anode over right M1 and cathode over left M1) vs. unilateral tDCS (with the anode over over right M1 and cathode over left supraorbital region) with fMRI was done in healthy volunteers (Sehm et al., 2012). Bilateral tDCS resulted in resting state changes in both primary and secondary motor areas, as well as in the prefrontal cortex, while unilateral M1 stimulation (with the anode over right M1 and cathode over the left supraorbital region) only influenced prefrontal, parietal, and cerebellar areas. Using seed-based connectivity metrics with a seed in the stimulated right M1, Sehm et al. (2013) showed that bilateral tDCS resulted in increased intracortical connectivity with right M1 after stimulation, which did not occur with unilateral stimulation. Both bilateral and unilateral tDCS resulted in decreased interhemispheric connectivity, however. This suggests that while tDCS over bilateral M1 (e.g., anode over left M1, cathode over right M1) increases connectivity within and between primary motor regions of the stimulated hemisphere, unilateral tDCS stimulation of only one hemisphere (e.g., anode over M1, cathode over a supraorbital region) only increases connectivity with other regions, such as parietal cortex and cerebellum. Some studies started to examine the effects of tDCS over other cortical areas, such as the left DLPFC. Results from these investigations are to some extent contradictory and require further exploration (Keeser et al., 2011; Pena-Gomez et al., 2012).

In summary, tDCS applied over a specific region induces distant effects on network connectivity, which may conceivably impact behavior. Modulation of distant neural regions via location-specific stimulation holds intriguing possibilities. However, caution is urged when interpreting these preliminary results, since within this handful of studies, there is great variability in the experimental designs used (e.g., in the stimulation montage, period of stimulation, recording method, time of recording, type of analysis performed). In addition, there is significant interindividual variability in results depending on the state of the subject's or network's activity (state-dependency), and the task performed. Evaluation of connectivity effects of tDCS in clinical populations may contribute to the understanding of behavioral deficits in these patients (for example, O'Shea et al., 2014). To this end, there is a need for studies that examine connectivity effects of tDCS in stroke patients at different time points (acute, subacute, chronic), with different lesion locations (cortical, subcortical), and with different levels of impairment.

It is possible that new NIBS stimulation paradigms using time-varying waveforms, periodical as in the case of alternating current stimulation (tACS) (Herrmann et al., 2013), or random as for random noise (tRNS) (Terney et al., 2008) may contribute in the future to more effective neurorehabilitative efforts. Preliminary studies show similar modulation of excitability in the sensorimotor cortices (Kanai et al., 2008; Feurra et al., 2011) and on cognitive functions (Polania et al., 2012a; Cappelletti et al., 2013; Santarnecchi et al., 2013) using these techniques.

While tDCS influences neuronal firing rates in a bimodal manner depending on its polarity, tACS seems to up- and down-regulate the firing rate affecting neuronal spike timing (Reato et al., 2010). tACS generates an alternating current at a specific frequency, with the potential to synchronize or desynchronize activity between targeted brain regions. tACS follows models of phase-locking communication and communication through coherence that suggest that neural populations communicate through time-locked oscillations (Fries, 2005), making it a potential way to modulate neural communication across brain regions. Such a feature may be used for tailoring individualized interventions aimed at coupling or decoupling activity between specific brain regions depending on the subject/patient if this is proved at some point to be desirable or therapeutically useful.

In contrast, tRNS involves the application of alternating currents at different, random frequencies to the scalp. Due to its oscillatory, rather than direct current, nature, it has been proposed that tRNS ensures the application of stimulation is polarity-independent (i.e., neither anodal or cathodal; Miniussi et al., 2013). High-frequency tRNS (100–640 Hz) has been shown to elicit powerful cortical excitability modulations with even longer after-effects than tDCS, reaching 70 min following 10 min of stimulation (Chaieb et al., 2011). These newer methods provide promising new ways to modulate excitability in the brain both locally and across neural networks.

## NIBS and cortical reorganization after stroke

Following stroke, patients with the most successful recovery of motor function are those whose patterns of brain activity as measured by fMRI most resemble those present in healthy volunteers (Johansen-Berg et al., 2002; Ward et al., 2003; Lotze et al., 2006; Nair et al., 2007; Grefkes and Fink, 2011). While healthy individuals show greater activity in the hemisphere contralateral to the hand they are moving, individuals with chronic stroke show in general a more bilateral pattern. Patients with greater motor impairment display increased fMRI activity in the contralesional hemisphere during attempted movement of the impaired hand (Johansen-Berg et al., 2002; Ward et al., 2003; Fridman et al., 2004; Lotze et al., 2006). In contrast, patients with better motor function show more normal patterns of ipsilesional motor activity, similar to the patterns one might see in healthy controls (Ward et al., 2003; Rehme et al., 2011). However, it is unclear which patients could benefit more from contralesional activity, if it serves an adaptive role (see for example Lotze et al., 2006).

Given these neuroimaging patterns after stroke, it has been proposed that upregulation of activity in the ipsilesional M1 or downregulation in the contralesional M1 might contribute to improved motor control (Ward and Cohen, 2004). Numerous proof of principle studies have now been done with some reporting that increasing excitability in ipsilesional M1 through high-frequency rTMS or anodal tDCS may yield improvements in motor performance or motor learning in healthy subjects (for example, Nitsche et al., 2003a,b,c; Reis et al., 2009) and small clinical studies have demonstrated modest, yet variable, improvements in individuals with stroke (Hummel and Cohen, 2005, 2006; Khedr et al., 2005; Kim et al., 2006; Pomeroy et al., 2007; for a review, see Sandrini and Cohen, 2013). Importantly for rehabilitation, it has been proposed that some of these changes outlast the period of stimulation (Khedr et al., 2010; Krawczyk, 2012).

Similarly, downregulating excitability in the contralesional motor cortex in chronic stroke patients was also associated with improvements in motor function, along with increased cortical motor excitability in the ipsilesional M1 and decreased cortical excitability in the contralesional M1 (Fregni et al., 2005; Takeuchi et al., 2005, 2008). Consistently, low-frequency rTMS or cathodal tDCS applied to downregulate excitability in the contralesional hemisphere resulted in motor gains. When applied for this purpose, single sessions of 10–25 min of rTMS over the contralesional M1 were reported to induce improvements in movement kinematics (Mansur et al., 2005; Takeuchi et al., 2005; Boggio et al., 2006; Liepert et al., 2007; Dafotakis et al., 2008; Nowak et al., 2008). When applied over several days, with or without motor training, some improvements were reported in grip strength and upper extremity function as measured by the Fugl-Meyer score and other assessments (Kirton et al., 2008; Kakuda et al., 2011).

It is also possible to use simultaneous stimulation of the ipsilesional cortex, with inhibition of the contralesional M1. This appears to also produce motor gains when combined with physiotherapy which last for 1 week (Lindenberg et al., 2010), but which seem to plateau after 2 weeks (Lindenberg et al., 2012). Bilateral stimulation over M1 with constraint-induced movement therapy also led to reported functional gains in the Fugl-Meyer test and handgrip strength. However, one recent study compared the differences between anodal, cathodal, and bilateral stimulation in stroke patients and demonstrated that anodal and cathodal stimulation had greater effects on motor output (via MEPs) than bilateral stimulation (O'Shea et al., 2014). Moreover, the effects of high-frequency rTMS over M1 may be more pronounced in individuals with subcortical, compared to cortical, stroke, suggesting that different patients may be differentially susceptible to beneficial effects of these techniques (Ameli et al., 2009).

To this end, it should be kept in mind that there is by no means agreement on the extent or universality of these beneficial effects and that well-controlled multicenter clinical trials are required to assess this issue (Wallace et al., 2010; Talelli et al., 2012). Further research should be done to determine the most effective paradigms for brain stimulation and to factor in the lesion location, specific genetic markers if any (e.g., BDNF), levels of motor or cognitive impairment or neuroimaging patterns as predictors of responsiveness to NIBS. More insight into this topic and great caution is required until results from well-designed multicenter clinical trials are available (Ridding and Rothwell, 2007; Kandel et al., 2012; Rothwell, 2012; Sandrini and Cohen, 2013).

## Future directions for NIBS research in neurorehabilitation

NIBS represents a novel and exciting tool to modulate cortical excitability, in specific local and distant brain regions and has been shown to alter connectivity with areas interconnected with the stimulated site. One exciting new application of NIBS is this ability to modulate functional connectivity between different interconnected regions and its proposed impact on behavior. For instance, dual-site stimulation paradigms, such as paired pulse stimulation applied repetitively could potentially modulate connectivity between two specific regions (Buch et al., 2011).

Another line of research is based on the ability of NIBS to modulate brain intrinsic oscillatory activity as in the framework of brain-computer interface applications (Soekadar et al., 2011), through the use of frequency-specific entrainment (Thut et al., 2012). To this effect, paradigms can be designed to enhance or decrease activity within the range of physiologically-relevant, region-specific frequencies, for instance, resonance phenomena with endogenous brain rhythms. Newer methods of brain stimulation, such as tACS and tRNS mentioned previously, may also prove useful toward this effort.

A recent feasibility study demonstrated that it was possible to combine tDCS with MEG recording, and in addition, provide a chronic stroke patient with neurofeedback about her brain activity in motor regions in the form of a visual stimulus and a robotic orthosis that opened and closed as her hand moved (Figure 3; Soekadar et al., 2013a,b, 2014a,b). This preliminary work showed that stimulation with online neural recording and feedback was feasible in the MEG environment, and results in enhanced performance after stimulation. The use of NIBS with other forms of brain-computer interfaces, robotic prosthetics, or with enhancement of pharmacological treatment may yield greater gains, due to the influence of NIBS by specific neurotransmitters as mentioned previously.

The use of NIBS in conjunction with other methods like neuroimaging or genetic analyses may prove particularly useful, not only to study what NIBS does to distributed brain activity, but also to identify predictors of response to NIBS interventions. For instance, O'Shea et al. (2014) used MR spectroscopy and behavioral measures to identify who responders to tDCS interventions. They found that GABA concentration in the stimulated region could predict the magnitude of behavioral changes after anodal tDCS.

Finally, emerging combinations of new methods are afforded by improvements in technology, computing, and mathematical modeling, such as simultaneous tDCS stimulation with MEG recordings (Soekadar et al., 2013a,b). NIBS in conjunction with biofeedback training designed to help individuals control their own brain activity may also contribute to neurorehabilitation (Buch et al., 2012). Using these methods in conjunction with brain-computer interfaces, virtual reality displays, or other feedback paradigms may contribute new insights to improve neurorehabilitative efforts using NIBS.

### Conflict of interest statement

The authors declare that the research was conducted in the absence of any commercial or financial relationships that could be construed as a potential conflict of interest.
